# Enhanced Absorption with Graphene-Coated Silicon Carbide Nanowires for Mid-Infrared Nanophotonics

**DOI:** 10.3390/nano11092339

**Published:** 2021-09-08

**Authors:** Patrick Rufangura, Iryna Khodasevych, Arti Agrawal, Matteo Bosi, Thomas G. Folland, Joshua D. Caldwell, Francesca Iacopi

**Affiliations:** 1 School of Electrical and Data Engineering, Faculty of Engineering and IT, University of Technology Sydney, Broadway, NSW 2007, Australia; Patrick.Rufangura@student.uts.edu.au (P.R.); iryna.khodasevych@uts.edu.au (I.K.); Arti.Agrawal@uts.edu.au (A.A.); 2 Australian Research Council Centre of Excellence on Transformative Meta-Optical Systems, School of Electrical and Data Engineering, Faculty of Engineering and IT, University of Technology Sydney, Broadway, NSW 2007, Australia; 3 IMEM-CNR, Parco Area delle Scienze 37/A, 43124 Parma, Italy; matteo.bosi@imem.cnr.it; 4 Department of Physics and Astronomy, The University of Iowa, Iowa City, IA 52242, USA; thomas-folland@uiowa.edu; 5 Department of Mechanical Engineering, Vanderbilt University, Nashville, TN 37212, USA; josh.caldwell@vanderbilt.edu

**Keywords:** phonon-plasmon hybridization, subwavelength confinement, epitaxial graphene, silicon carbide nanowires, mid-infrared nanophotonics

## Abstract

The mid-infrared (MIR) is an exciting spectral range that also hosts useful molecular vibrational fingerprints. There is a growing interest in nanophotonics operating in this spectral range, and recent advances in plasmonic research are aimed at enhancing MIR infrared nanophotonics. In particular, the design of hybrid plasmonic metasurfaces has emerged as a promising route to realize novel MIR applications. Here we demonstrate a hybrid nanostructure combining graphene and silicon carbide to extend the spectral phonon response of silicon carbide and enable absorption and field enhancement of the MIR photon via the excitation and hybridization of surface plasmon polaritons and surface phonon polaritons. We combine experimental methods and finite element simulations to demonstrate enhanced absorption of MIR photons and the broadening of the spectral resonance of graphene-coated silicon carbide nanowires. We also indicate subwavelength confinement of the MIR photons within a thin oxide layer a few nanometers thick, sandwiched between the graphene and silicon carbide. This intermediate shell layer is characteristically obtained using our graphitization approach and acts as a coupling medium between the core and outer shell of the nanowires.

## 1. Introduction

The mid-infrared (MIR) range of the electromagnetic (EM) spectrum hosts various molecular vibrational fingerprints [1,2], making it an exciting spectrum for photonic applications [3,4]. For instance, the MIR detectors are understood to be very important in sensing applications such as exhaled breath detection [5], water-quality monitoring [6], cancerous tissue diagnosis [7], and greenhouse gas detection [8]. Nanophotonics offers the possibility to improve infrared technology significantly [9,10]. Surface plasmon polariton (SPP) modes result from strong coupling of bound EM fields with collective charge oscillations (plasmon) in a conductor, enabling subwavelength manipulation of light and light–matter interactions. Graphene is one of the promising plasmonic materials that can excite strongly confined SPPs in the MIR and terahertz (THz) spectral ranges with remarkable dynamic tunability and electric field confinement, unrealizable with conventional metal plasmonics [9,11,12]. To date, several graphene-based novel photonic devices such as optical modulators [13], photodetectors [14,15], switches [16], antennas [17], waveguides [18], sensors [19], and polarizers [20] have been proposed.

On the other hand, polar dielectric materials such as BN, SiO_2_, and SiC simultaneously support low loss and sub-wavelength confined EM fields in the MIR and THz spectral range via the stimulation of surface phonon polariton (SPhP) modes [21]. SPhPs arise from the strong interaction of EM waves and collective vibration of ionic lattice within the Reststrahlen band, a narrow spectral range between transverse (TO) and longitudinal optic (LO) phonon frequencies. The ability to confine MIR wavelengths through excitation of SPPs and SPhPs has many implications for optics and optoelectronic technologies such as MIR photodetection [22], sensing [23], and photovoltaics cells [24]. Optical devices that combine graphene and polar materials are inferred to significantly advance MIR photonic technologies due to the exceptional polaritonic responses resulting from the hybridization of SPP and SPhP modes in these material systems. For example, recent studies of hybrid SPP-SPhP modes in graphene on polar materials revealed the resultant dispersion relation to be significantly modified [25,26].

Thanks to the advancement of graphene synthesis techniques, the 2D material can now be grown on, or transferred to, and characterized with different polar dielectric substrates [27]. However, in most cases, the transfer of graphene onto the substrate deteriorates graphene’s properties and is limited to small-scale samples. Epitaxial graphene (EG) grown on silicon carbide is a suitable platform for growing graphene directly on semiconductors for diverse technologies and applications [27,28]. Over the past few years, some theoretical and experimental works were optically conducted on graphene on bulk silicon carbide [25,29,30,31]. Moreover, our recent study demonstrated strong confinement and significant propagation figures of merit (FOM) for hybrid SPP-SPhPs in graphene on silicon carbide [32]. Uniform quality EG can now be grown over a large scale on silicon carbide on silicon [27,33,34], which offers the opportunity to develop and characterize complex graphene/silicon carbide 3D structures at the wafer- scale with nanoscale precision, using advanced silicon processing technologies. EG can also be grown conformally on curved SiC surfaces, such as nanoparticles and nanowires [35,36], forming core-shell nanostructures.

The addition of a shell consisting of a different material to a metal nanoparticle has been shown in some cases to enhance absorption and broaden the tunability range of the localized plasmon resonance by adjusting the shell’s thickness. Several two-layered systems, such as metal nanoparticles with dielectric shell [37] or Si core with metal shell [38], were demonstrated for optical wavelengths. While metal-dielectric-metal [39,40] or Si-dielectric-metal [41] configurations, which are of particular interest for biomedical applications [42], have been studied well, fewer examples of three-layered multi-shell nanostructures have been reported. There is great interest in three-layered nanostructures as it has generally been shown that the addition of a dielectric spacer layer drastically changes the electric field distribution within the structures, opening the possibility for subwavelength confinement and enhancement of the field within the spacer [40,41].

An analogy can be drawn between metal nanoparticles in the optical regime and SiC phonon polariton response within the Reststrahlen band at the MIR range. A few initial studies on SiC-graphene nanoparticles have been published [43,44], but only limited theoretical research was conducted on curved multilayer metal-graphene [45,46] or more general SiC-graphene nanostructures [47,48]. Hence there is a need for further research to investigate MIR characteristics of core-shell SiC-graphene nanoparticles and nanowires, particularly now that a clear path exists to obtain such structures experimentally.

In this work, we combine experiments and electromagnetic simulations based on the finite element method to demonstrate MIR photon absorption enhancement in EG-coated silicon carbide nanowires (3C-SiC NWs). EG was conformally grown on 3C-SiC NWs using a catalytic alloy mediated approach [36], which naturally also leads to the formation of a thin oxide (silicon oxycarbide) layer between the graphene and the SiC core. The numerical simulations and experimental data point towards the hybridization between the surface phonon and the surface plasmon polaritons in the SiC/graphene core/shell nanosystem, with oxide acting as a coupling medium. The proposed materials platform results hold promise for various MIR photonic applications, such as molecular sensing and other applications that require strong field and absorption enhancement.

## 2. Materials and Methods

### 2.1. Numerical Simulations

**Figure 1:**
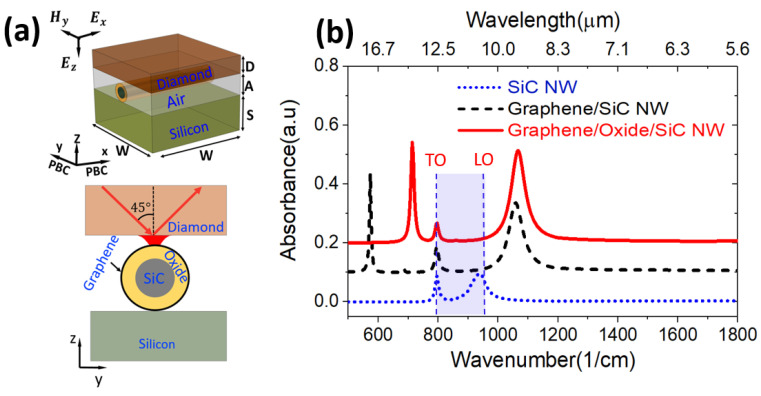
(**a**) Schematic of the graphene/oxide/SiC NW simulation model in 3D view (upper graph) and cross-section view (lower graph), (**b**) The simulated absorbance for SiC NW, Graphene/SiC NW, and Graphene/Oxide/SiC NW models. W = 500 nm, S = 200 nm, A = 120 nm, D = 100 nm. The total diameter of NW is 50 nm consisting of the oxide shell thickness of 4 nm and SiC diameter of 42 nm.

Numerical simulations to investigate the MIR responses of the NWs were performed in COMSOL Multiphysics [49] using the finite element method (FEM). A schematic of the simulated model is shown in Figure 1a. We modelled a single NW of a total 50 nm diameter, consisting of a SiC core diameter of 42 nm and an oxide shell thickness of 4 nm, replicated at a regular pitch of W = 500 nm by applying periodic boundary conditions (PBC) in x and y coordinates. The pitch size was chosen to minimize cross-wire interactions.

The model utilizes a TM/p polarised wave with the plane of incidence along the wires incident at $\vartheta_{i}=45{}^{\circ}$ into the diamond (D = 100 nm) with a refractive index $n_{d}\approx 2.4$ in order to match the ATR-FTIR experimental conditions. An evanescent field with dominant vertical E_z_ component and negligible Ex or Ey components is excited in the air gap (A = 120 nm) after total internal reflection from the air-diamond interface, which couples to the surface plasmon and phonon in graphene and SiC, respectively.

The frequency-dependent complex permittivity measured on our 3C-SiC membrane sample was used as input to the simulation model for the material properties of SiC. The following parameters were extracted from the fit of the measured dielectric function to Lorentz oscillator/TO LO formalism with equations and fit graphs provided in the Supplementary Materials (Figure S2a): high-frequency permittivity $\varepsilon_{\infty }=6.52$, TO and LO phonon frequencies $\omega_{TO\;=}\;797{\;\mathrm{cm}}^{-1},\;\omega_{LO\;=}\;973{\;\mathrm{cm}}^{-1}\;$, and damping constant $\gamma =12{\;\mathrm{cm}}^{-1}$.

The graphene was modelled as a 2D conductive layer using a transition boundary condition (TBC). TBC enables simulating an ultra-thin conductive layer as an impedance boundary without requiring a mesh across the thickness of the material. The TBC uses the thickness and dielectric properties of a material (refractive index/permittivity) as input and computes transfer and surface impedances of the thin conductive layer to establish a connection between the surface current on both sides of the boundary [49]. The following parameter values were used for calculated permittivity of graphene with Equation (S2) provided in the Supplementary Materials (Figure S2b): Fermi energy $E_{F}=0.37$ eV, equivalent to the carrier concentration of $1\times 10^{13}\;{\mathrm{cm}}^{-2}$ as measured with Hall Effect [34], relaxation time τ=370 fs, and T=300 K. Silicon (S= 200 nm) was treated as a non-dispersive medium with a refractive index $\;n_{s}=3.42$. The oxide layer between graphene and SiC NW is a silicon oxycarbide (SiOC) with a refractive index n = 1.5 [50]. Absorption A = 1-R-T was calculated from reflectance R and transmittance T and is shown in Figure 1b. We also built additional intermediate models for SiC NW/Si and graphene/SiC NW/Si with total wire diameter of 50 nm in all cases (also graphene/air/Si in Supplementary Materials Figure S3).

### 2.2. Graphene Growth and Experimental Characterization

The bare 3C-SiC NWs were grown on Si (100) substrates at the IMEM-CNR Institute, Italy, using a chemical vapor deposition reactor at 1100 °C, with nickel nitrate [Ni (NO_3_)_2_] and carbon monoxide use as a catalyst and gaseous precursor, respectively. Note that the 3C-SiC NW growth temperature of 1100 °C together with the presence of oxygen and nickel may lead to a sporadic and incomplete graphitization of the wires surface during their growth [36,51]. In this work, we have investigated two batches of SiC nanowires with different extents of unintended graphitization, the first with barely any (bare SiC nanowires) and the second one with a higher extent of graphitization (weakly graphitized).

The graphitization of 3C-SiC NWs was performed using a Ni/Cu catalytic alloy mediated growth using the SiC nanowires as a solid source of carbon [33,36]. Thanks to the liquid phase epitaxial growth nature of this synthesis technique and hence the long and efficient adatom diffusion, the graphene grown via this process can conformally coat the nanowires. In addition, a highly oxidized (silicon oxycarbide) layer that is a few nanometers thick is simultaneously formed in between graphene layers and SiC [36,52]. More details about the growth of 3C-SiC NWs on Si substrates were reported in [53], and details of graphitization are reported in the Supplementary Materials (Figure S1).

Surface morphology characterization of the NWs samples before and after graphitization was performed using a Zeiss Supra 55 VP high-resolution emission scanning electron microscope operated at 2 kV.

Raman spectroscopy of the nanowires was performed with a Renishaw inVia Raman microscope activated by a 633 nm laser. The measurements were conducted on nine different spots on samples sized 1 cm ×1 cm, and the D and G and 2D spectral intensities were recorded.

The IR spectroscopy of the samples was performed at room temperature with a Thermo Scientific Nicolet 6700 using an attenuated total reflectance Fourier transformed infrared (ATR-FTIR) spectrometer. The diamond crystal with 45° of the incident radiation was used for the ATR system. The samples’ absorption spectra were collected using 100 scans, and the average intensity was reported in arbitrary units (a.u).

## 3. Results

### 3.1. Simulation Results

A schematic of the SiC/graphene core/shell nanowires is shown in Figure 1a. The model consists of SiC NW coated with graphene on Si substrate, including a silicon oxycarbide (SiOC) layer sandwiched between the graphene and the SiC core. The simulated absorbance of a bare SiC NW (blue dotted line in Figure 1b) revealed two weak absorption peaks at 797 cm^−1^ and 940.5 cm^−1^. The first corresponds to the SiC’s bulk transverse optical (TO) phonon mode. The second peak, which appears within the SiC Reststrahlen window (shown in blue shading), is attributed to a Fröhlich/localized dipole surface phonon polariton (LSPhP) mode, which occurs when condition ε_SiC_ = −2ε_Air_ is satisfied, as explained further in Section 3.1.1.

The simulation conducted with graphene-coated SiC NW, without considering an oxide layer in between (black dashed line in Figure 1b), showed the splitting of the modes resulting in three absorption peaks: a sharp absorption at ~ 574 cm^−1^, plus the SiC’s bulk TO mode, and another broader peak at 1060 cm^−1^. The splitting of the peaks results from the hybridization of graphene’s SPP mode with SiC localized dipole SPhP mode in a core-shell type configuration, resulting in localized phonon-plasmon coupled modes M1 and M2.

The simulated results on the graphene/oxide/SiC NW model (red solid line in Figure 1b) show three absorption peaks at 714 cm^−1^ (M1), 797 cm^−1^ (TO mode), and at 1067 cm^−1^ (M2). The presence of oxide also leads to a blueshift of the modes. There is a higher contrast between the permittivity of SiC and oxide at the lower frequency mode than at the higher frequency mode. The weaker absorption of the TO peak, a bulk mode, is expected due to the reduction in the total volume of the SiC core from 50 nm to 42 nm diameter in the model.

#### 3.1.1. Modes and Electric Fields Analysis

Resonant frequencies of cylindrical nanostructures can be calculated as poles of electric polarizability derived from the electrostatic approximation of Lorenz–Mie theory [39,54,55]. The theory applies to both metallic and dielectric nanoparticles. Localized dipolar SPP and SPhP are considered due to the orientation of the incident evanescent vertical electric field perpendicular to the nanowires. While metal core-shell nanoparticles are represented with layers of finite thickness, graphene is usually treated as a conductive boundary due to its negligible thickness which allows adding conductivity term to the polarizability expression rather than increasing the number of layers. For a two-layer SiC/graphene core-shell nanoparticle, the resonant frequencies can be determined by setting the denominator of electric polarizability to zero [47,48]:

(1) $$\begin{array}{c} \alpha \left(\omega \right)=4\pi R^{3}\frac{\varepsilon_{SiC}\left(\omega \right)-\varepsilon_{air}+2g\left(\omega \right)}{\varepsilon_{SiC}\left(\omega \right)+2\varepsilon_{air}+2g\left(\omega \right)} \end{array}$$

(2) $$\begin{array}{c} g\left(\omega \right)=\frac{i\sigma \left(\omega \right)}{\varepsilon_{0}\omega R} \end{array}$$

where *R* is the total radius of the particle, $\varepsilon_{0}$ is the permittivity of free space, σ is graphene conductivity, $\varepsilon_{SiC}$ and $\varepsilon_{air}$ are the permittivities of SiC and surrounding air, correspondingly. SiC permittivity is frequency-dependent and can take positive and negative (metallic) values, so the resonance condition can be satisfied at multiple frequencies, representing hybridization of graphene’s shell SPP mode with SiC core SPhP mode and resulting in phonon-plasmon coupled modes M1 for lower frequency and M2 for higher frequency. The presence of a dielectric different to air, such as oxide, is known to shift plasmonic resonance in metallic nanoparticles; however, it does not lead to the appearance of new resonances. In the absence of graphene (*g* = 0), Equation (1) gives a Fröhlich resonance condition of a bare SiC nanoparticle, 940.5 cm^−1^.

**Figure 2:**
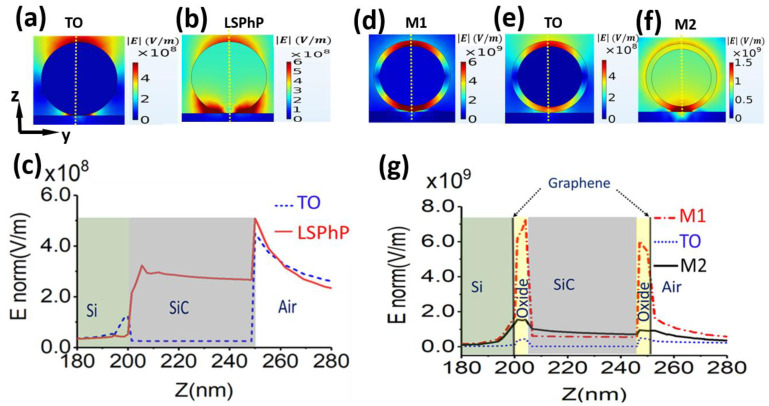
Simulated electric field distribution profiles and the magnitude of electric field intensities. (**a**,**b**) simulated electric field distribution profile on bare SiC NW for TO mode at 797 cm^−1^and LSPhP at 940.5 cm^−1^ showing the majority of field concentrated around the SiC NW surface, (**c**) magnitude of electric field intensity calculated along the cutline (yellow vertical dash lines in (**a**,**b**) on bare SiC NW for TO and LSPhP modes, (**d**–**f**) calculated electric field distribution profiles on graphene/oxide (4 nm)/SiC NW for M1 mode at 714 cm^−1^, TO mode at 797 cm^−1^ and M2 mode at 1067 cm^−1^ showing maximum fields concentrated inside the oxide layer (**g**) the calculated magnitude of electric field intensity along the cutline (yellow vertical dash lines in (**d**–**f**) for the graphene/oxide/SiC NW for M1 mode at 714 cm^−1^, TO mode at 797 cm^−1^ and M2 mode at 1067 cm^−1^ demonstrating strong enhancement of the field within the oxide shell layer.

Furthermore, we analyzed the electric field norm distribution at the resonances for bare SiC NW and graphene/oxide/SiC NW models. Figure 2a,b show the calculated electric field distribution (|E|) for bare SiC NW/Si at the resonances for TO mode and LSPhP (at 940.5 cm^−1^) where maximum electric fields are around the SiC NW surface. This is also shown by the magnitude of electric fields calculated along a cutline in the z-direction (yellow vertical dash lines in Figure 2a,b), showing a high electric field at the surface of the SiC NW exponentially decaying inside the SiC core (Figure 2c).

We subsequently calculated electric field norm distribution profiles of the graphene/oxide/SiC NW model for M1, TO, and M2 modes (Figure 2d–f). After introducing oxide between graphene and SiC NW, the electric field distribution became significantly confined within the oxide layer. This is explained by the calculated magnitude of electric fields along the cutline (yellow vertical dash lines in Figure 2d–f), showing the electric field increasing inside the oxide and exponentially dropping inside the SiC (Figure 2g). It is noticeable that the magnitude of electric field intensity in the graphene/oxide/SiC NW is one order of magnitude higher than the calculated electric fields on the bare SiC NW model (Figure 2c,g). Vector plots of the fields for M1 and M2 can also be found in Figure S5. As a comparison, the calculated field profiles for the M1, TO and M2 modes in the graphene/SiC nanowire without an intermediate oxide shell are shown in the Supplementary Material, Figure S4.

#### 3.1.2. The Roles of the Oxide Shell Thickness and Refractive Index on the MIR Response of the System

**Figure 3:**
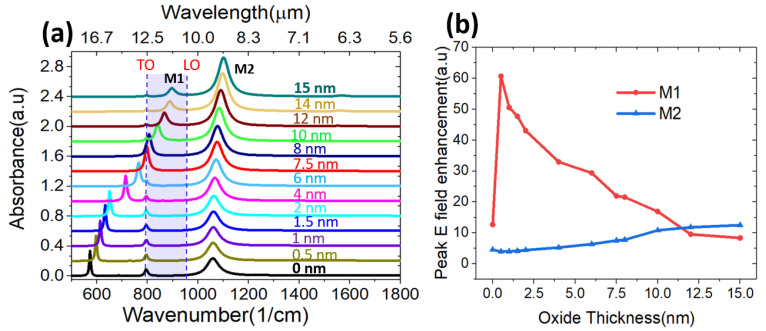
(**a**) Simulated absorbance of graphene -coated SiC nanowires at different oxide layer thicknesses, (**b**) The calculated peak field enhancement for different oxide shell thicknesses. Field enhancement was calculated at the peak absorption for each oxide shell thickness. Note that for an oxide shell thickness of zero, i.e., no oxide between the SiC core and the graphene outer shell, the M1 field enhancement drops dramatically, as well as for thicknesses over 10 nm.

As the experimental nanowire forest is found to have varying NW diameters hence also varying oxide thicknesses, we examined the effect of the oxide shell thickness on the system’s absorbance and electric field intensities. The simulations were performed for oxide shell thickness between zero (no oxide, only graphene shell) and 15 nm, while the NW’s total diameter was kept constant at 50 nm, i.e., the reduced size of the SiC core is compensated by oxide thickness. In Figure 3a, the simulated absorbance at different oxide layer thicknesses revealed a blue shift of the modes when the oxide thickness is increased. Also, the absorption intensity of M1 is weakened while the absorption intensity for M2 is enhanced. We also note that for an oxide thickness between ~6 and 10 nm, the M1 position falls in close proximity to the bulk TO mode, leading to complete overlap for an oxide shell of about 7–8 nm thickness and creating a misleading impression of an enhanced TO peak.

We subsequently calculated electric field enhancement for M1 and M2 using Equation (3):

(3) $$\begin{array}{c} E_{enhancement}=\frac{\left|E\right|}{\left|E_{0}\right|} \end{array}$$

where $E_{\;}$ represents the electric field calculated along the vertical cutline (yellow vertical line in Figure 2d–f) in the graphene/oxide/SiC NW model, while $E_{0}$ is the electric field calculated at the same point along the cutline without graphene/oxide/SiC NW in the model.

The calculated peak field enhancement showed a more substantial field enhancement of M1 than M2 (Figure 3b). The strongest peak field enhancement for M1 corresponds to oxide with a smaller thickness. Three scenarios occurred for M1 while varying the oxide thickness: under-coupling, optimal coupling, and over-coupling [56]. From Figure 3b, optimal coupling for M1 corresponds to an oxide thickness of ~1 nm, where a maximum peak field enhancement of about 60 is calculated. Below 1 nm, different physics might apply due to the 2D nature of the material. However, for an oxide shell thickness of zero, i.e., no oxide between the SiC core and the graphene outer shell, the M1 enhancement drops dramatically (over-coupling), as well as for thicknesses over 10 nm (under-coupling). It is also worth noting that the oxide layer appears to have a more dramatic influence on M1 than M2.

**Figure 4:**
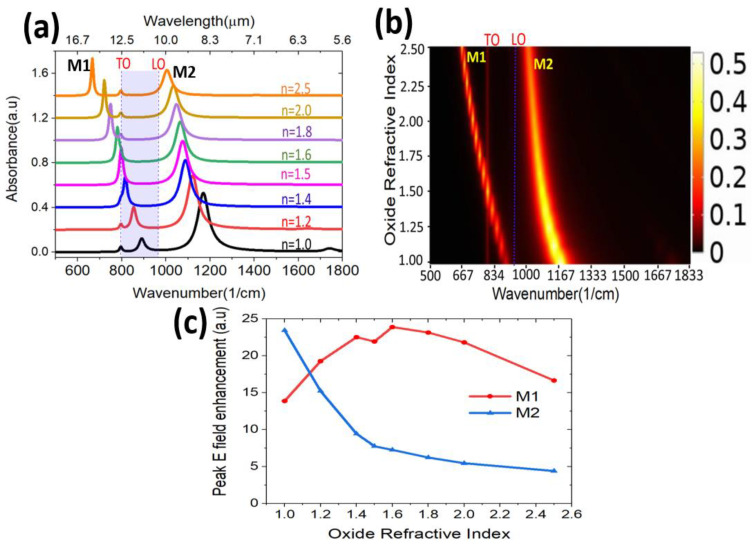
Simulated data of graphene/oxide/SiC NW at a different oxide’s refractive index, (**a**) absorbance spectra, (**b**) mode position and intensity for different oxide refractive index, (**c**) peak electric field enhancement.

In addition, we performed a sensitivity study by varying the oxide shell refractive index (n) and observing the effect on the MIR response of the graphene/oxide/SiC NWs. The shell of the graphitized 3C-SiC nanowires consists of a low-density SiOC medium [36,52], where the SiC has been gradually depleted of its carbon due to the solid-source graphitization process. Therefore, it is safe to assume that its refractive index would not be uniform through the entire shell. Hence, we performed the simulation by varying the refractive index of the oxide between 1 (air gap) and 2.5 (pure silicon carbide, as if no oxide was present between SiC and graphene). Figure 4a,b show the absorption spectra and profile map calculated for a fixed nanowire size of 50 nm diameter (thickness of oxide = 7.5 nm and diameter of SiC = 35 nm). Both M1 and M2 redshift, the absorption intensity of M1 is enhanced, while the intensity of M2 weakens when the oxide shell refractive index is increased.

We also analyzed the electric field enhancement at the resonance for different oxide refractive indexes using Equation (3). The calculation revealed the high field enhancement of ~24 for M1, corresponding to n ~1.6 (Figure 4c). This happens as a result of M1 being located closer to the TO frequency, where SiC exhibits the largest permittivity, which is vital for highly confined surface modes [57,58]. The calculated peak electric field enhancement for M2 revealed the highest enhancement of ~24 when n = 1 (air) is used for the shell between SiC and graphene, then dropping monotonically for higher refractive indexes.

### 3.2. Experimental Results and Discussion

**Figure 5:**
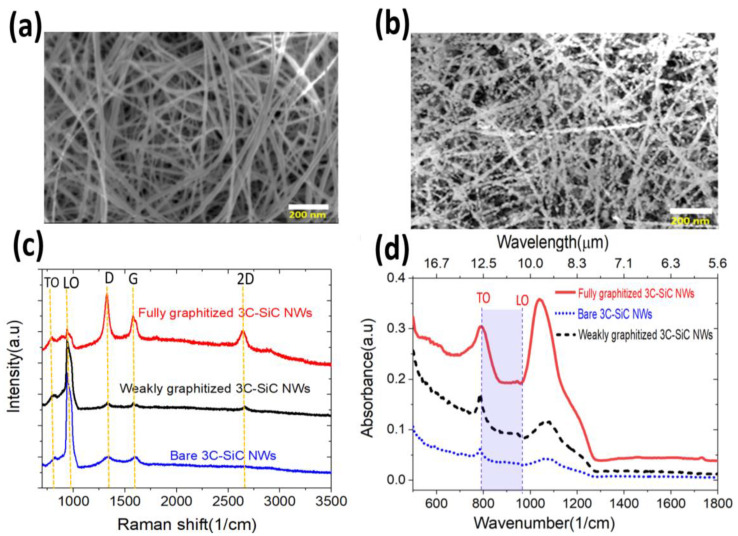
Morphology, Raman, and infrared characterization of the 3C-SiC NWs samples. SEM images of (**a**) bare and (**b**) graphitized 3C-SiC NWs. (**c**) The measured Raman spectra for bare 3C-SiC NWs, the weakly and fully graphitized 3C-SiC NWs. Note that the Raman spectra are intentionally offset for comparison. (**d**) The measured absorbance for bare 3C-SiC NWs, the weakly and fully graphitized 3C-SiC NWs. The highlighted spectral range between TO and LO represents the Reststrahlen band, where SiC can support a surface phonon polariton mode.

The scanning electron microscope (SEM) images of the bare and graphitized 3C-SiC NWs samples are shown in Figure 5a,b. The average diameter of as-grown 3C-SiC NWs varies between 30 nm and 50 nm (Figure 5a). More morphology characteristics along with energy-dispersive X-ray spectroscopy (EDS) and transmission electron microscope (TEM) data of as grown 3C-SiC NWs was reported in [36]. A decrease in the diameter of SiC NW and increased granularity is seen after graphitization as the nanowires are a solid source for the carbon in the graphene (Figure 5b). The diameter distributions of the nanowires estimated from the SEM images before and after graphitization are reported in the Supplementary Materials (Figure S7). The fully graphitized SiC NWs possess a shell of low-density oxide (silicon oxycarbide) medium between the graphene and SiC NWs. This is a direct result of the catalytic graphitization of 3C-SiC NWs and also occurs on flat surfaces [33,36,52]. The formed oxide medium has varying thicknesses due to the varying diameter of the as-grown bare SiC NWs, as shown by transmission electron microscope (TEM) data [36].

In Figure 5c, the Raman spectrum of bare SiC NWs revealed the TO and LO phonon modes at 797 cm^−1^ and 973 cm^−1^, respectively, which are the characteristics of 3C-SiC. The LO peak is convoluted with the Raman response from the silicon substrate, as expected [36,51,59]. Extremely weak peaks around the positions of the D and G bands of graphene are observed for the bare 3C-SiC NWs, showing a negligible extent of unintended graphitization of the wires. This effect is more prominent on the second batch of the as -grown SiC nanowires, which henceforth we refer to as “weakly graphitized”. The Raman spectrum of the weakly graphitized NWs shows, in addition, a faint fingerprint of the 2D peak at 2684 cm^−1^.

Fully (and intentionally) graphitized 3C-SiC NWs revealed prominent D, G, and 2D bands at 1340 cm^−1^, 1578 cm^−1^, and 2684 cm^−1^, respectively, matching the characteristic of epitaxial graphene on 3C-SiC on Si [33]. However, a large average D over G band intensity ratio (I_D_/I_G_) of 1.13 as compared to the I_D_/I_G_ of ~0.2 in the normal epitaxial graphene grown on flat 3C-SiC on Si samples [34], is a characteristic of the graphitized SiC NWs due to their small diameter of 30–50 nm.

The IR absorption characteristics of the as-grown and graphitized SiC NWs are reported in Figure 5d.

**Figure 6:**
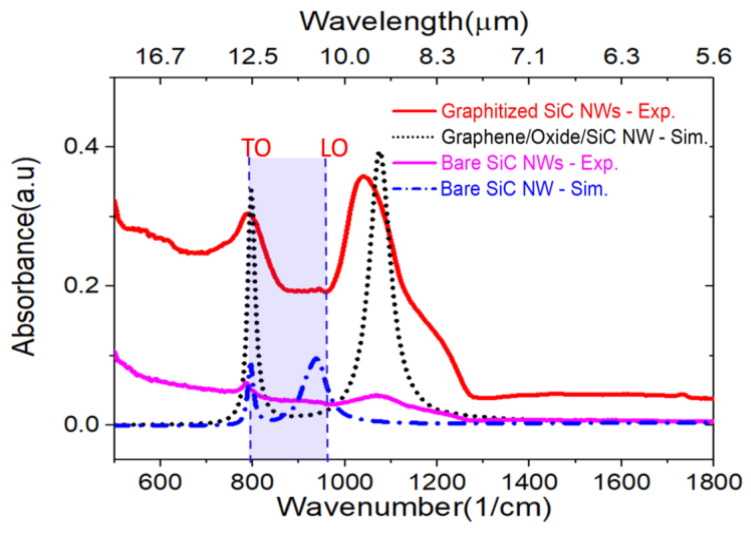
A comparison of the measured IR absorbance spectral of bare and fully graphitized 3C-SiC NWs and the simulated absorbance on bare SiC NW, and graphene/oxide/SiC NW with an oxide shell thickness of 7.5 nm and refractive index of 1.5.

A comparison of the experimental data from Figure 5d and simulated results for oxide thickness =7.5 nm (red curve from Figure 3a) is shown in Figure 6. Both experimental and simulated absorbance of the bare SiC nanowires show low IR absorption with a weak but sharp absorption peak at ~797 cm^−1^_,_ corresponding to the 3C-SiC TO mode. The simulated LSPhP mode at ~940.5 cm^−1^ is weak and broad in measured absorbance spectra on the bare SiC nanowires sample, making it unlikely to be observed in our experimental forest of nanowires. The weak broad absorption measured around 1050 cm^−1^ cannot be attributed to a phonon-polariton as it is outside of the SiC Reststrahlen band and hence is attributed to vibrational resonances of hydroxyls and epoxy functional groups [60,61] stemming from the oxide in the outer shell of the as-grown SiC nanowires [36,53]. The IR absorption spectra of the weakly graphitized NWs (Figure 5d) show similar fingerprints but with an increased absorbance.

The experimental IR spectrum of fully graphitized SiC nanowires reveals a substantial five to nine-fold enhancement for the two measured absorption modes, as compared to the bare SiC nanowires. Interestingly, one of the experimental modes seems to overlap with the SiC TO, around 797 cm^−1^. An absorption enhancement of the bulk TO mode can be excluded, as in fact the total volume size of SiC nanowires is expected to decrease after graphitization because of the consumption of the SiC, a solid source for the catalytic graphitization, while the oxide outer shell in between the SiC and the graphene is increased to a typical value of ~8–10 nm as observed experimentally with a transmission electron microscope [36,53]. From the simulated spectra in Figure 3a, oxide shells roughly in the 6–10 nm thickness range would lead effectively to the overlap of TO and M1 modes (see also Figure S8 for absorption of SiC at different nanowire diameters). Simulated oxide shell thickness of 7.5 nm and a refractive index of 1.5, corresponding to a generic silicon oxycarbide medium [50] were chosen for comparison of the simulation and experiment. Therefore, we suggest that there is an overlap between the TO and M1 responses. This all indicates that surface polariton phenomena are at work in this material system.

The second mode at around 1050 cm^−1^ from the measured absorbance on the fully graphitized SiC nanowires is particularly broad and shows a significant enhancement. While we expect that the hybridized mode M2 would correspond to the experimental position of this second mode, as shown by the simulated spectrum in Figure 6, it is worth noting that here we are likely to observe a convoluted response caused by the vibrational resonances of epoxy-functional groups present in the nanowires sample. In addition, the fact that those groups are physically concentrated in the oxide shell between the SiC core and the graphene outer shell would likely lead to further amplification of their IR fingerprint, thanks to the field concentration taking place in the oxide, as shown in the electrical fields in Figure 2g.

The regular simulated pattern with minimal cross-talk between wires (Figure 1a) is clearly highly simplified with respect to the highly irregular experimental NWs forest in the SEM images (Figure 5a,b), with random wire pitch, orientation, and varying diameters (see distribution in Supplementary Material, Figure S7). However, the simplified model provides enough fundamental information to interpret the more complex response from the experimental forest of nanowires. The key differing aspects are those of varying spatial orientation, varying dimensions and the presence of cross-wire interactions. The response of randomly oriented nanowires effectively provides a combined response to irradiation by incident waves with electric field orientation both along and across the wires. Note that the predominantly vertical E_z_ field leads to similar dipolar responses for either orientation of incident electric field (plane of incidence either along or across the wires), except that the TO peak is absent when there is no field component along the wires (as shown in the Supplementary Material, Figure S6). The experimental size distribution is expected to introduce a strong peak broadening (see Figure S8 for spectra simulated for different nanowire sizes), as well as the additional cross-wire interactions that were minimized in the numerical model, but are not expected to change the fundamental nature of the response.

## 4. Conclusions

We have shown an experimental nine-fold absorption enhancement in graphene-coated SiC nanowires as compared to the absorption of as-grown, bare SiC nanowires. With the help of finite element multiphysics modelling, we attribute this effect to the hybridization of the surface phonon in SiC and the surface plasmon response in graphene. Furthermore, we revealed this hybridization to generate mode splitting and extend the graphitized nanowires’ spectral resonances beyond the SiC’s Reststrahlen band.

We also show that the oxide layer between the graphene shell and the SiC core acts as a coupling medium, leading to a substantial absorption and electric field enhancement. We indicate an extreme subwavelength confinement for a MIR wavelength around ~10 µm within a few nanometers (<10 nm) of the oxide shell medium. Additionally, the model indicates that a more ordered and periodic nanowire structure could potentially lead to even higher enhancement factors up to 60 times, and we indicate that this effect can also be used to enhance the IR fingerprint of molecules physically trapped in the intermediate shell (epoxy molecules, in this case). We believe that these findings open up a promising avenue towards tunable MIR molecular sensors and detectors based on core/shell 3C-SiC/graphene nanostructures.

## Data Availability

Data is contained within the article or Supplementary Materials.

## Supplementary Materials

The following are available online at https://www.mdpi.com/article/10.3390/nano11092339/s1, Figure S1: Schematic demonstrating a catalytic graphitization process for epitaxial graphene growth on cubic silicon carbide (3C-SiC) nanowires on a silicon substrate, Figure S2: Dielectric function of SiC and graphene used in our simulations. (**a**) A comparison between measured permit-tivity in 3C-SiC and the calculated permittivity using the TOLO model showing the best fit between measurement and calculations when ω_TO_ = 797 cm^−1^, ω_LO_ = 973 cm^−1^, ε_ꝏ_= 6.52, and γ = 12 cm^−1^ used as input parameters in Equation (S1). (**b**) The calculated permittivity of graphene using Equation (S2). Graphene was simulated as a monolayer with a thickness of 0.33 nm, E_F_ = 0.37 eV, and τ = 370 fs. Figure S3: Electromagnetic simulation model set up. (**a**) Schematic of four different simulated models: Bare SiC NW, graphene/air, graphene/SiC NW, and graphene/oxide/SiC NW. (**b**) Simulated absorbance for four different models: bare SiC NW, graphene/air, graphene/SiC NW, and graphene/oxide/SiC NW. The following geometry parameters were used: W = 500 nm, S = 200 nm, A = 120 nm, D = 100nm. For all four simulations, the total nanowire diameter is 50 nm. For the graphene/oxide/SiC NW model, oxide shell thickness was 4 nm while SiC was 42 nm, Figure S4: Simulated electric field profiles and maps for graphene on SiC nanowires with no intermediate oxide layer. (**a**–**c**) Simulated electric field maps for modes M1 at 574 cm^−1^, TO at 797 cm^−1^and M2 at 1060 cm^−1^, respectively. (**d**) The magnitude of electric field intensity calculated along the cutline (yellow vertical dash lines in **a**,**b**) on graphene/SiC NW for M1, TO and M2 modes, Figure S5: Electric field vectors for (**a**) M1 and (**b**) M2 in graphene/oxide/SiC NW model when oxide shell thickness is 4 nm., Figure S6: Absorbance for different orientations of the incident field for 4nm thick oxide layer (**a**) Hy, Ex, Ez (**b**) Hx, Ey, Ez. The Ez component in both cases dominates the response. Lack of electric field component along the wire (Ex) significantly reduces TO response in (**b**), Figure S7: Nanowires diameter distribution as estimated from SEM data of bare and graphitized 3C-SiC NWs samples using Image J software. (**a**) Bare 3C-SiC NWs, the average diameter is 48.6 nm (**b**) fully graphitized 3C-SiC NWs, the average diameter is 44.8 nm, Figure S8: Effect of the diameter of SiC on the absorption and field enhancement of graphene/oxide/SiC NW model. (**a**) Simulated spectra absorption and (**b**) color profile map showing the spectra absorption profile at different SiC diameters, (**c**) peak field enhancement at different SiC diameters. The simulation was performed by varying the diameter of SiC while the oxide thickness and refractive index were kept fixed at 7.5 nm and 1.5, Figure S9: Dynamic tunability analysis for M1 and M2 in graphene/oxide/SiC NW. (**a**) The simulated absorptions spectra showing a blue shift effect on M1 and M2 when the Fermi energy (E_F_) in graphene is increased, (**b**) color profile map showing the spectra absorption profile at different graphene’s Fermi energy E_F_, (**c**) peak field enhancement for different Fermi energy showing high peak field enhancement of ~26 for M1 and ~10 for M2.
