# Supplementary material for: Enhanced Absorption with Graphene-Coated Silicon Carbide Nanowires for Mid-Infrared Nanophotonics
Source: Nanomaterials (Basel). 2021 Sep 8;11(9):2339. doi: 10.3390/nano11092339 (PMC8465231; doi:10.3390/nano11092339)
Supplement: Supplementary file 1 [file nanomaterials-11-02339-s001.zip › nanomaterials-1345520-supplementary.pdf]

nanomaterials

MDPI

Supplementary Materials

Enhanced Absorption with Graphene-Coated Silicon Carbide Nanowires for Mid-Infrared Nanophotonics

Patrick Rufangura [1,2], Iryna Khodasevych [1,2], Arti Agrawal [1,2], Matteo Bosi [3], Thomas G. Folland [4], Joshua D. Caldwell [5] and Francesca Iacopi [1,2,*]

1 School of Electrical and Data Engineering, Faculty of Engineering and IT, University of Technology Sydney, Broadway, NSW 2007, Australia; Patrick.Rufangura@student.uts.edu.au (P.R.); iryna.khodasevych@uts.edu.au (I.K.); Arti.Agrawal@uts.edu.au (A.A.)
2 Australian Research Council Centre of Excellence on Transformative Meta-Optical Systems, School of Electrical and Data Engineering, Faculty of Engineering and IT, University of Technology Sydney, Broadway, NSW 2007, Australia
3 IMEM-CNR, Parco Area delle Scienze 37/A, 43124 Parma, Italy; matteo.bosi@imem.cnr.it
4 Department of Physics and Astronomy, The University of Iowa, Iowa City, IA 52242, USA; thomas-folland@uiowa.edu
5 Department of Mechanical Engineering, Vanderbilt University, Nashville, TN 37212, USA; josh.caldwell@vanderbilt.edu
* Correspondence: francesca.iacopi@uts.edu.au

1. Growth Parameters

The bare 3C-SiC NWs were grown on Si (100) substrates using a chemical vapor deposition reactor at 1100 °C, using nickel nitrate [Ni $(NO_3)_2$] and carbon monoxide as a catalyst and gaseous precursor, respectively. Using a cryopump deposition chamber with a vacuum pressure of ~10^{-5} mbar and deposition current of 200 mA, a double blanket layer of Nickel (Ni ~5 nm) and copper (Cu ~10 nm) was sputtered on 3C-SiC NWs/Si substrates. Ni/Cu/3C-SiC NWs/Si samples were then annealed at a high temperature of ~1050 °C (25 °C/min) for an hour under the medium vacuum condition of ~10^{-5} mbar and then gradually cooled down to room temperature. Note that no final wet etch was performed on the graphitized nanowires to remove residual metal catalysts and excess carbon, due to the low adhesion of the nanowires to their substrate.

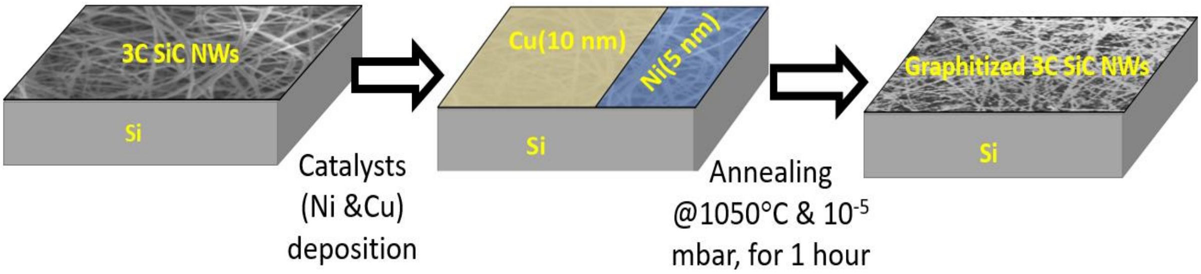

Figure S1. Schematic demonstrating a catalytic graphitization process for epitaxial graphene growth on cubic silicon carbide (3C-SiC) nanowires on a silicon substrate.

2. SiC and Graphene Material Properties

The frequency-dependent permittivity of SiC and graphene used as input to the model are provided in Figure S2a,b. The dielectric function data of our 3C-SiC can also be reproduced using a Lorentz oscillator/TOLO formalism:

$$\varepsilon(\omega) = \varepsilon_\infty \left(1 + \frac{\omega_{Lo}^2 - \omega_{To}^2}{\omega_{To}^2 - \omega^2 - i\gamma\omega}\right) \quad \text{(S1)}$$

where ε_∞ represents the high-frequency permittivity while ω_{TO} and ω_{LO}, symbolize the TO and LO phonon frequencies correspondingly, and γ describes the damping constant associated with optic phonon mode.

The effective dielectric function of graphene $\varepsilon_g(\omega)$ was calculated using Equation (S2):

$$\varepsilon_g(\omega) = \varepsilon_0 + \frac{i\sigma(\omega)}{\omega\Delta} \quad \text{(S2)}$$

where $\sigma(\omega)$, ε_0 and Δ represent frequency-dependent conductivity of graphene, the permittivity of free space, and the effective thickness of graphene, respectively. The conductivity of graphene was modelled using the well-known Kubo formalism and is expressed by the sum of interband (σ_{intra}) and intraband (σ_{inter}) transitions [1].

$$\sigma_{intra} = \frac{2ie^2T}{\pi\hbar^2(\omega + i\tau^{-1})} ln\left[2cosh\left(\frac{E_F}{2T}\right)\right] \quad \text{(S3)}$$

$$\sigma_{inter} = \frac{e^2}{4\hbar}\left[\frac{1}{2} + \frac{1}{\pi}arctan\left(\frac{\hbar\omega - 2E_F}{2T}\right) - \frac{i}{2\pi} ln\frac{(\hbar\omega + 2E_F)^2}{(\hbar\omega - 2E_F)^2 + (2T)^2}\right] \quad \text{(S4)}$$

$$\sigma(\omega) = \sigma_{intra} + \sigma_{inter} \quad \text{(S5)}$$

where k_B and $\hbar$ are the Boltzmann and reduced Plank constant, respectively, e is an elementary charge of an electron, E_F Fermi energy and τ is relaxation time, temperature $T = 300\,K$.

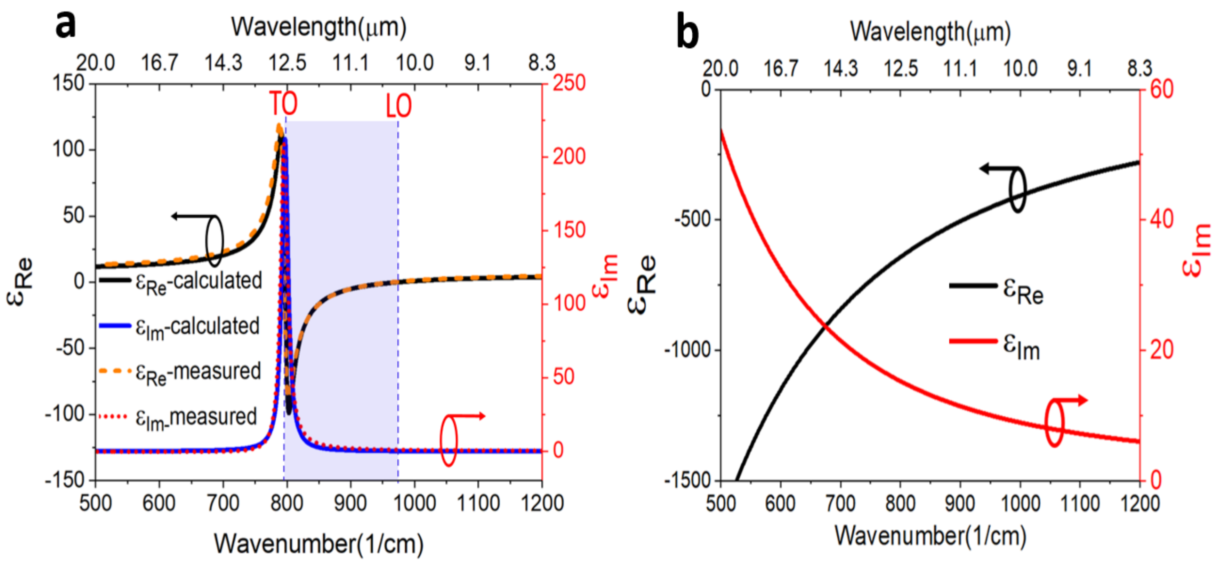

Figure S2. Dielectric function of SiC and graphene used in our simulations. (**a**) A comparison between measured permittivity in 3C-SiC and the calculated permittivity using the TOLO model showing the best fit between measurement and calculations when $\omega_{TO=}$ 797 cm^{-1} and $\omega_{LO=}$ 973 cm^{-1}, ε_{∞} = 6.52, and γ = 12 cm^{-1} used as input parameters in Equation (S1). (**b**) the calculated permittivity of graphene using Equation (S2). Graphene was simulated as a monolayer with a thickness of 0.33nm, E_F = 0.37 eV, and τ = 370 fs.

3. Additional Simulation Models

Four models were built and investigated, as shown in Figure S3a. We first built a SiC NW/Si model, graphene/air/Si, followed by a graphene/SiC NW/Si model, and then finally, we created a graphene/oxide/SiC NW/Si model. The simulated absorbance for four different models are shown in Figure S3b.

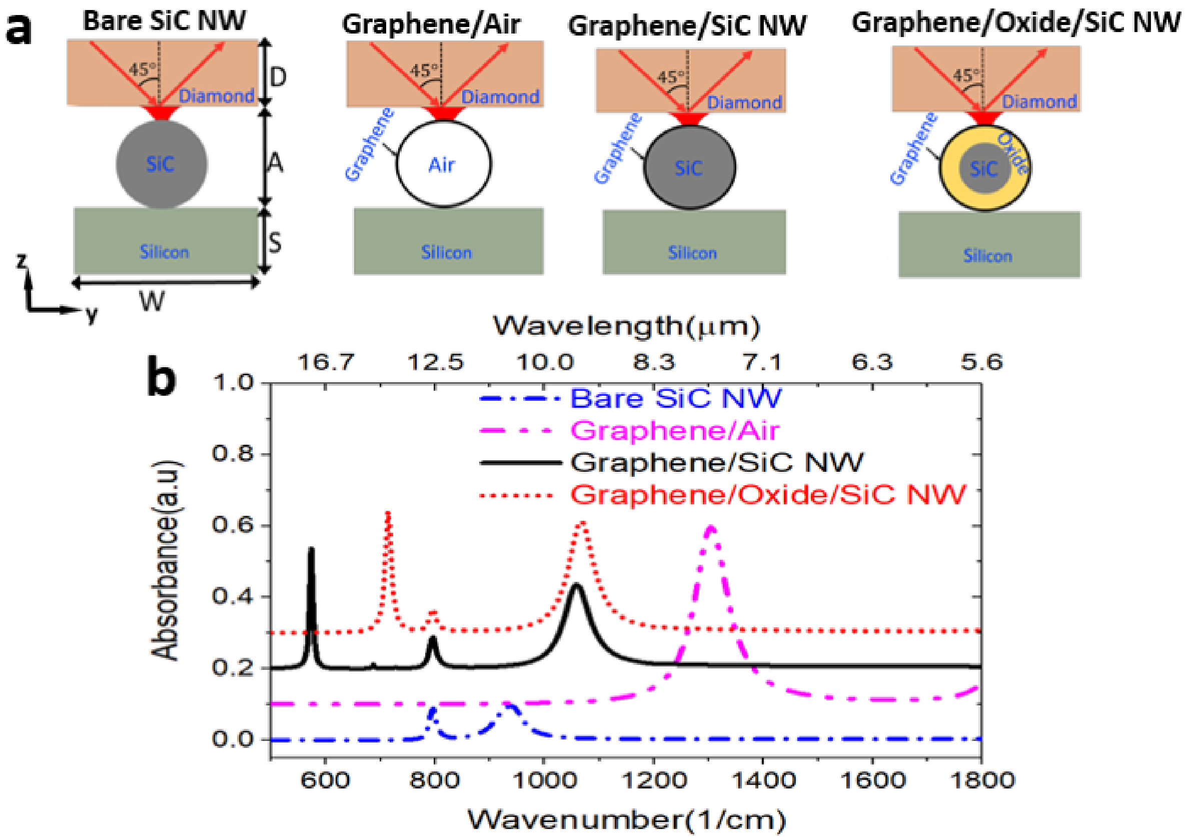

Figure S3. Electromagnetic simulation model set up. (**a**) Schematic of four different simulated models: Bare SiC NW, graphene/air, graphene/SiC NW, and graphene/oxide/SiC NW. (**b**) Simulated absorbance for four different models: bare SiC NW, graphene/air, graphene/SiC NW, and graphene/oxide/SiC NW. The following geometry parameters were used: W = 500 nm, S = 200 nm, A = 120 nm, D = 100nm. For all four simulations, the total nanowire diameter is 50 nm. For the graphene/oxide/SiC NW model, oxide shell thickness was 4 nm while SiC was 42 nm.

4. Electric Field Distribution Profiles and the Magnitude of Electric Field Intensities for when There Is No Oxide Layer between the Graphene Shell and the SiC Core

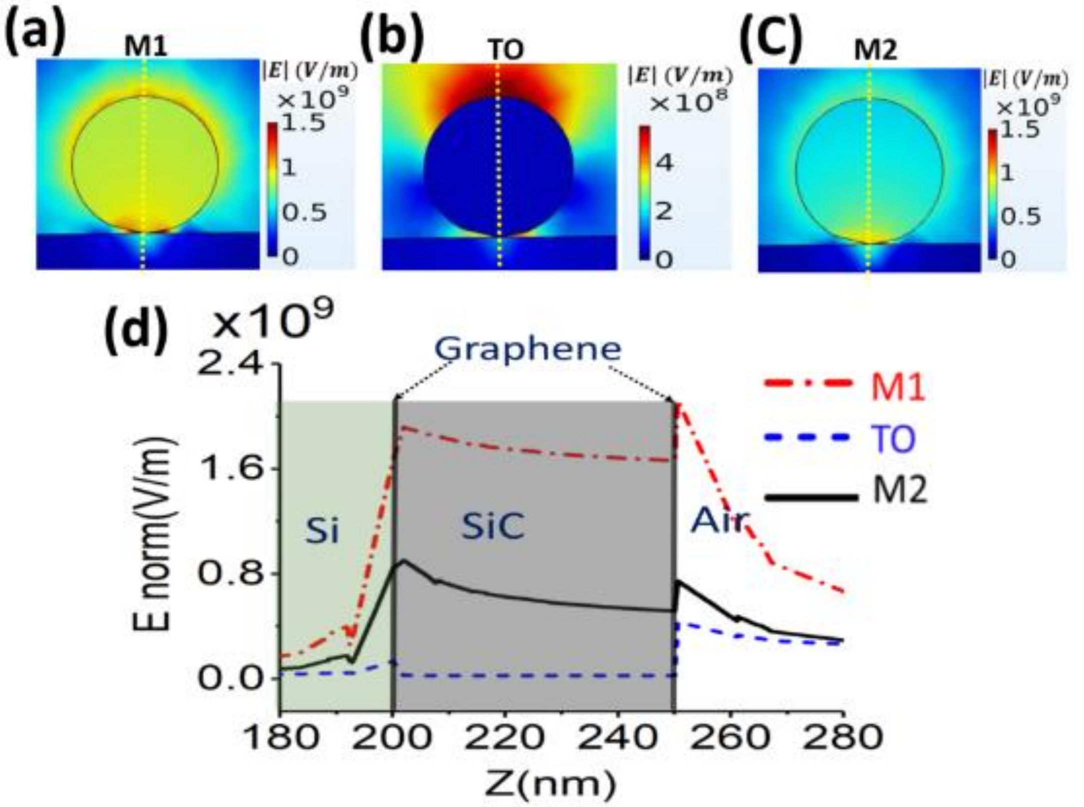

Figure S4. Simulated electric field profiles and maps for graphene on SiC nanowires with no intermediate oxide layer. (**a**–**c**) Simulated electric field maps for modes M1 at 574 cm^{-1}, TO at 797 cm^{-1}and M2 at 1060 cm^{-1}, respectively. (**d**) The magnitude of electric field intensity calculated along the cutline (yellow vertical dash lines in **a**,**b**) on graphene/SiC NW for M1, TO and M2 modes.

5. Electric Field Vector Analysis for M1 and M2 in Graphene/Oxide/SiC NW Model

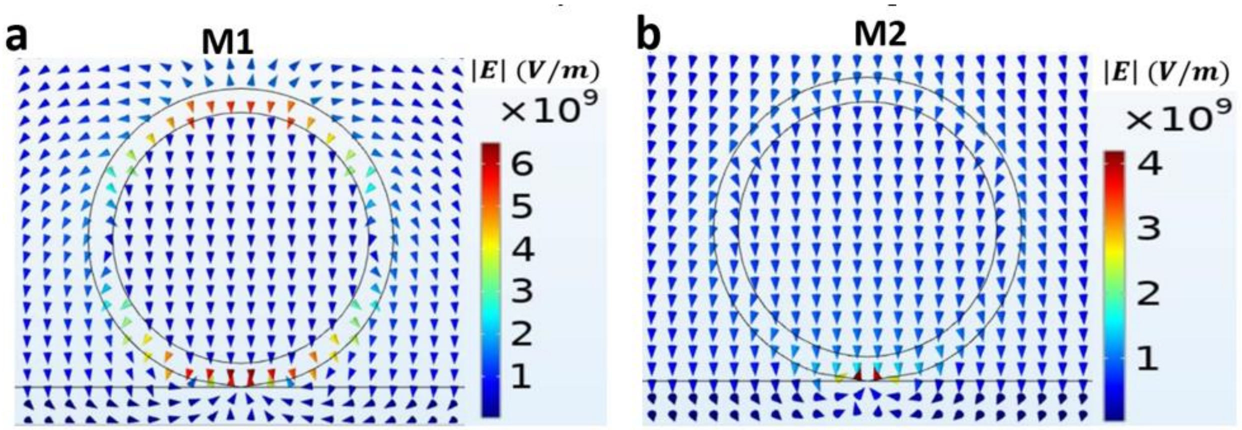

Figure S5. Electric field vectors for (**a**) M1 and (**b**) M2 in graphene/oxide/SiC NW model when oxide shell thickness is 4 nm.

6. Simulated Absorbance for Different Orientations of the Incident Fields/TM Polarized Wave's Incident

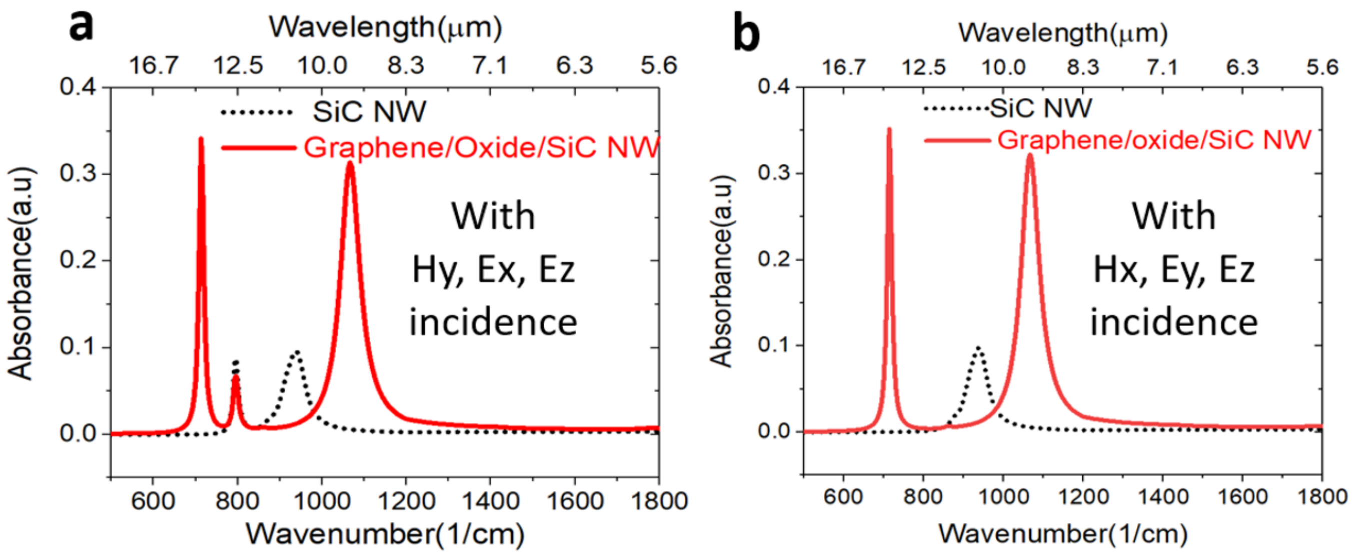

Figure S6. Absorbance for different orientations of the incident field for 4nm thick oxide layer (**a**) Hy, Ex, Ez (**b**) Hx, Ey, Ez. The Ez component in both cases dominates the response. Lack of electric field component along the wire (Ex) significantly reduces TO response in (**b**).

7. Nanowires Diameter Distributions

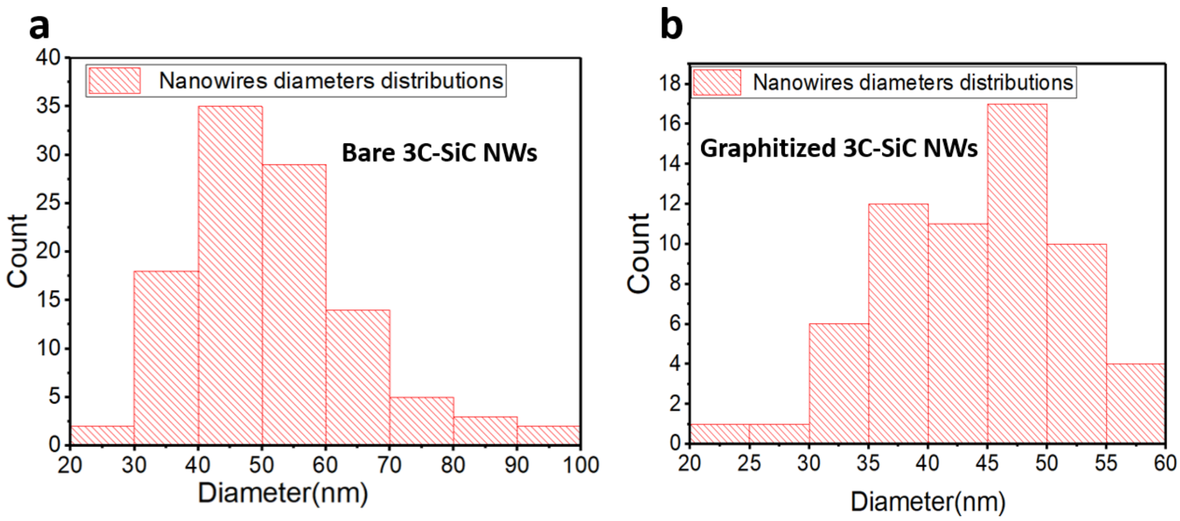

Figure S7. Nanowires diameter distribution as estimated from SEM data of bare and graphitized 3C-SiC NWs samples using Image J software [2]. (**a**) Bare 3C-SiC NWs, the average diameter is 48.6 nm (**b**) fully graphitized 3C-SiC NWs, the average diameter is 44.8 nm.

8. Effect of the Diameter of SiC on the Absorption and Electric Field Enhancement

We performed a sensitivity study by varying the diameter of SiC and recording the effect on the MIR response of the graphene/oxide/SiC NWs. We increased the diameter of SiC from 10 nm to 90 nm and kept oxide shell thickness fix to 7.5 nm. The simulated absorption spectra and profile map at different SiC diameters are shown in Figure S8a,b. The simulated absorbance revealed a redshift effect for both M1 and M2 when the diameter of SiC is increased. For a smaller diameter of 10 nm, the TO mode is not noticeable in the absorbance spectra as this is a bulk mode the intensity of which is entirely dependent on

the size of SiC. We also noticed that the intensity of M1 becomes very weak for smaller SiC diameters and disappears for diameters below 20 nm, while it is enhanced for larger SiC diameters.

Moreover, we calculated the electric field enhancement for different SiC diameters. As shown in Figure S8c, the calculated peak electric field enhancement increases with increasing SiC diameter and reaches the highest enhancement of ~27 when the diameter of SiC is ~50 nm and dropping monotonically for diameters >50 nm. For M2, the field enhancement is higher for smaller SiC diameters, with the highest enhancement of 22 calculated for SiC diameter of 10 nm, while electric field enhancement drops significantly for larger SiC diameters.

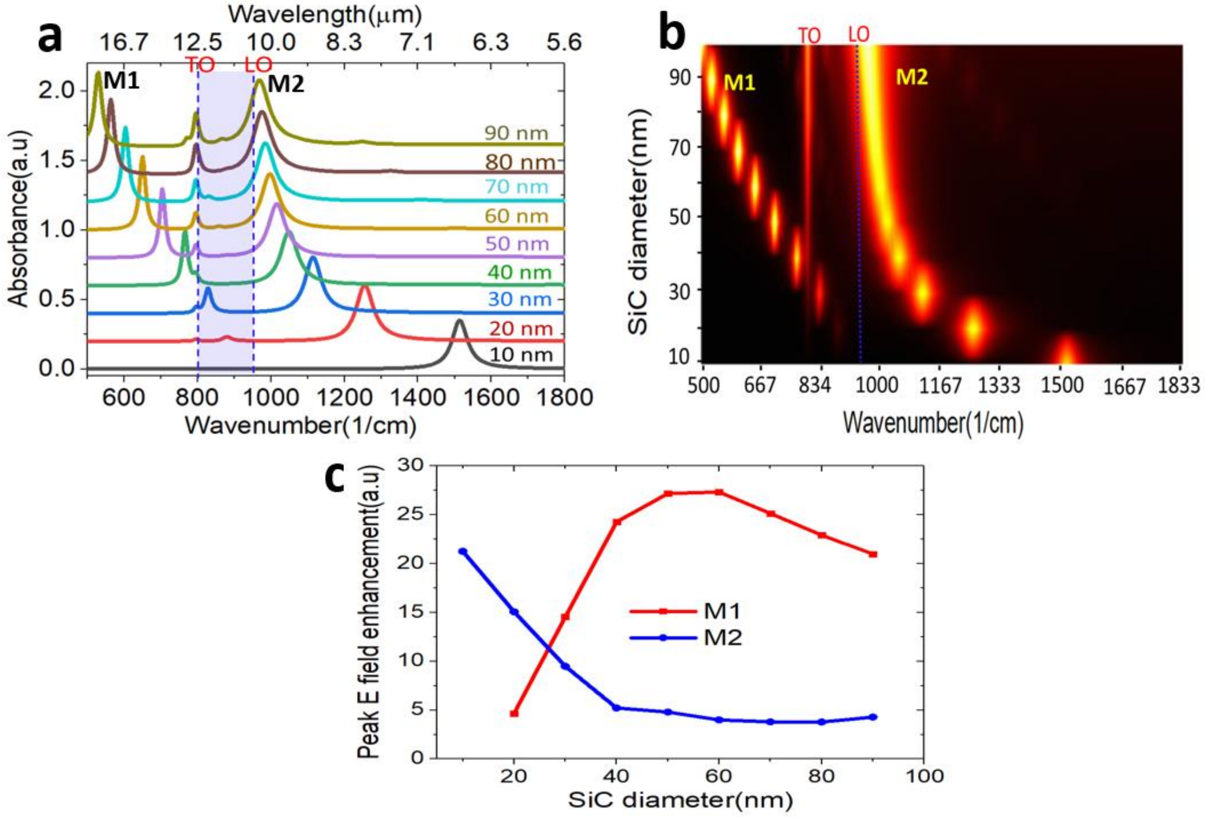

Figure S8. Effect of the diameter of SiC on the absorption and field enhancement of graphene/oxide/SiC NW model. (**a**) Simulated spectra absorption and (**b**) color profile map showing the spectra absorption profile at different SiC diameters, (**c**) peak field enhancement at different SiC diameters. The simulation was performed by varying the diameter of SiC while the oxide thickness and refractive index were kept fixed at 7.5 nm and 1.5.

9. Dynamic Tunability Analysis

Among the exciting properties of graphene is its electrostatic gate tunability of the carrier concentrations/Fermi energy which makes the SPP modes supported in this material dynamically tunable. The carrier concentrations in graphene can be increased from ~10^{11} cm^{-2} to 10^{13} cm^{-2}as a consequence of the Dirac fermions linear dispersion in this material [3]. To investigate the dynamic tunability of the modes in our graphene/oxide/SiC NW system, we fixed the diameter of nanowires to 50 nm (thickness of oxide = 7.5 nm and diameter of SiC = 35 nm) and refractive index of oxide to 1.5. We also assumed a constant relaxation rate $\tau = 370\ fs$ in Equation (S5). Then we performed the simulation at varying Fermi energy (E_F) ranging from 0.212 eV to 0.40 eV according to the reported value of carrier concentrations in EG on flat 3C-SiC/Si [4]. The simulated absorbance spectra and the absorption profile map showing the tunability of M1 and M2 in graphene/oxide/SiC NW are shown in Figure S9a,b. A blueshift effect of ~150 cm^{-1} for M1 and ~128 cm^{-1} for

M2 was realized by increasing graphene's Fermi energy from 0.212 eV to 0.4 eV. Moreover, the calculated E-field enhancement at the resonance frequencies for different E_F revealed up to ~26 enhancement of the field for M1 corresponding to graphene's E_F of ~0.3 eV (Figure S9c). It was also noticed that the E field for M2 is enhanced as the E_F increases with maximum field enhancement of ~10 realized when the E_F of graphene is 0.4 eV.

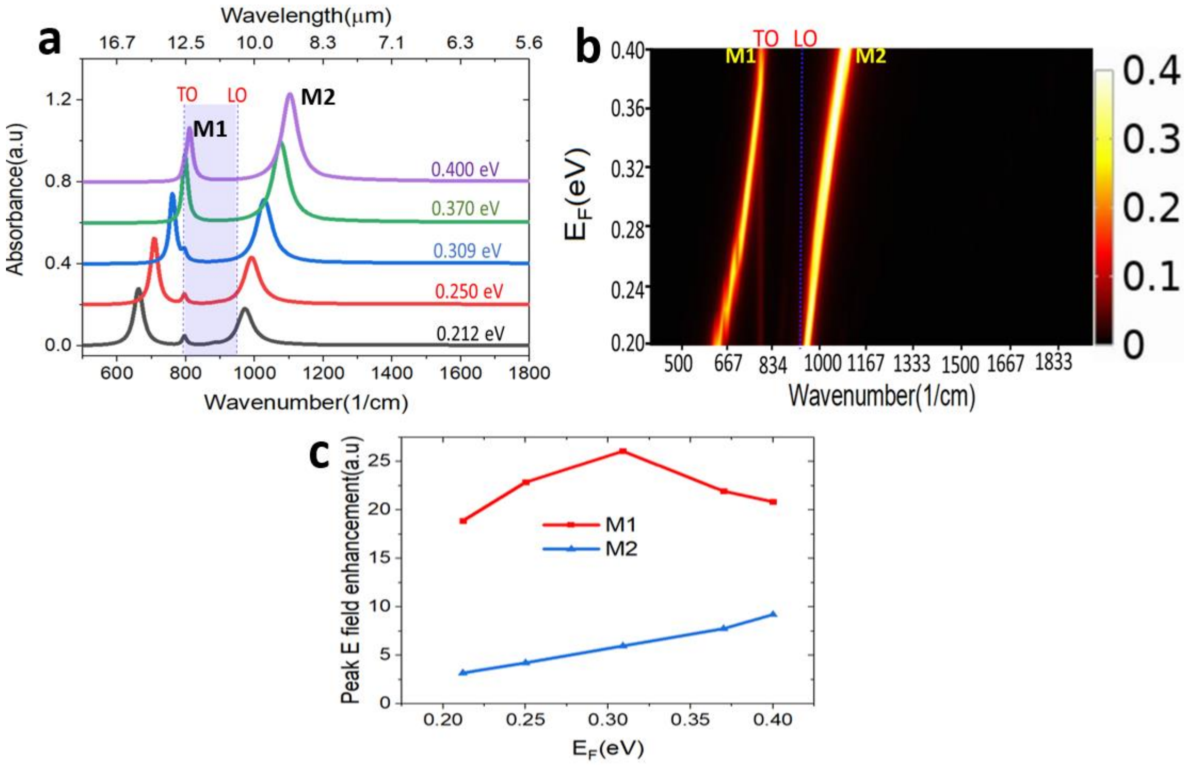

Figure S9. Dynamic tunability analysis for M1 and M2 in graphene/oxide/SiC NW. (**a**) The simulated absorptions spectra showing a blue shift effect on M1 and M2 when the Fermi energy (E_F) in graphene is increased, (**b**) color profile map showing the spectra absorption profile at different graphene's Fermi energy E_F, (**c**) peak field enhancement for different Fermi energy showing high peak field enhancement of ~26 for M1 and ~10 for M2.

References

1. Francescato, Y.; Giannini, V.; Maier, S.A. Strongly confined gap plasmon modes in graphene sandwiches and graphene-on-silicon. *New J. Phys.* **2013**, *15*, 063020.
2. Schneider, C.A.; Rasband, W.S.; Eliceiri, K.W. NIH Image to ImageJ: 25 years of image analysis. *Nat. Methods* **2012**, *9*, 671–675.
3. Low, T.; Avouris, P. Graphene plasmonics for terahertz to mid-infrared applications. *ACS Nano* **2014**, *8*, 1086–1101.
4. Pradeepkumar, A.; Amjadipour, M.; Mishra, N.; Liu, C.; Fuhrer, M.S.; Bendavid, A.; Isa, F.; Zielinski, M.; Sirikumara, H.I.; Jayasekara, T.; et al. p-Type Epitaxial Graphene on Cubic Silicon Carbide on Silicon for Integrated Silicon Technologies. *ACS Appl. Nano Mater.* **2019**, *3*, 830–841.
